# Endometrial tuberculosis increases miscarriage risk after IVF/ICSI compared with non-specific chronic endometritis

**DOI:** 10.1186/s12884-026-08960-2

**Published:** 2026-03-21

**Authors:** Juan Zou, Jia-Liang He, Lijuan Fu, Yubin Ding, Qi Wan

**Affiliations:** 1 Department of Pathology, West China Second University Hospital, Sichuan University, Chengdu, 610041 China; 2 Key Laboratory of Birth Defects and Related Diseases of Women and Children, Ministry of Education, West China Second Hospital, Sichuan University, Chengdu, 610041 China; 3 Department of Obstetrics and Gynecology, Women and Children’s Hospital of Chongqing Medical University, Chongqing, 401147 China; 4 Joint International Research Laboratory of Reproduction and Development of the Ministry of Education of China, School of Public Health, Chongqing Medical University, Chongqing, 401147 China; 5 Department of Obstetrics and Gynecology, the First Affiliated Hospital of Chongqing Medical University, Chongqing, 401147 China; 6 Department of Pharmacology, Academician Workstation, Changsha Medical University, Changsha, 410013 China; 7 Department of Obstetrics and Gynecology, The Reproductive Medical Center, West China Second University Hospital, Sichuan University, Chengdu, 610041 China

**Keywords:** Endometrial tuberculosis, Non-specific chronic endometritis, In vitro fertilization, Intracytoplasmic sperm injection-embryo transfer, Miscarriage

## Abstract

**Background:**

Chronic endometritis (CE) has two principal subtypes—non-specific chronic endometritis (NSCE) and endometrial tuberculosis (ETB). Both can adversely affect embryo implantation. Although ETB is generally more severe, its low incidence has limited comparative data on assisted reproductive outcomes versus NSCE. A direct comparison of pregnancy outcomes following in vitro fertilization (IVF) or intracytoplasmic sperm injection-embryo transfer (ICSI-ET) between patients with ETB and those with NSCE would help clarify whether differential management strategies are warranted and provide more precise guidance for clinical practice.

**Methods:**

In this single-center retrospective study, clinical data were collected from infertile patients who underwent IVF/ICSI-ET at West China Second University Hospital, Sichuan University, between January 2019 and December 2024. All patients were diagnosed via hysteroscopic endometrial biopsy. ETB was verified through histopathology along with positive nucleic acid testing for the Mycobacterium tuberculosis complex, whereas NSCE was diagnosed by histopathology in combination with CD138 immunohistochemical staining.

**Results:**

A total of 409 patients were included: 27 in the ETB group and 382 in the NSCE group. No significant differences were observed between the two groups in age, duration of infertility, baseline FSH level, or number of embryos transferred (*P* > 0.05). The clinical pregnancy rate was comparable between the ETB group (51.9%) and the NSCE group (49.0%) (*P* > 0.05). However, the live birth rate was numerically lower in the ETB group (30.0%) than in the NSCE group (42.4%), though this difference was not statistically significant (*P* > 0.05). A significant difference was found in the miscarriage rate between the two groups with the ETB group having a higher miscarriage rate (*P* < 0.05). Multivariate regression analysis identified ETB as an independent risk factor for miscarriage after IVF/ICSI-ET.

**Conclusions:**

In patients undergoing IVF/ICSI-ET, ETB is associated with a significantly higher risk of miscarriage compared to NSCE, despite similar clinical pregnancy rates. This underscores the need for distinct clinical management and counseling in ETB cases.

**Supplementary Information:**

The online version contains supplementary material available at 10.1186/s12884-026-08960-2.

## Background

Chronic endometritis (CE) is defined by persistent endometrial inflammation, primarily involving plasma cell infiltration. It encompasses two principal subtypes: non-specific chronic endometritis (NSCE) and endometrial tuberculosis (ETB), While both subtypesconverge on the pathological endpoint of impaired endometrial receptivity but diverge etiologically, histologically, and clinically, necessitating distinct diagnostic and therapeutic approaches. NSCE is characterized by the presence of plasma cells in the endometrial stroma [1]. Although often asymptomatic or presenting with subtle symptoms such as abnormal uterine bleeding, pelvic discomfort, dyspareunia, and leukorrhea, NSCE is highly prevalent among infertile women and has been recognized as a significant factor in recurrent implantation failure (RIF) and recurrent pregnancy loss [2]. Diagnosis typically relies on histopathological examination with detection of CD138-positive plasma cells [3]. Hysteroscopy also plays an important role, revealing characteristic features such as focal or diffuse peri-glandular hyperemia, endometrial stromal edema, endometrial thickening, surface irregularity, and micropolyps [4]. The prevalence of NSCE has been reported to be as high as 57.8% in women with unexplained infertility [5].

ETB, a form of female genital tuberculosis, remains a common cause of chronic pelvic inflammatory disease and infertility in developing countries. ETB can lead to structural and functional alterations in the endometrium, adversely affecting embryo implantation and pregnancy maintenance. Research indicates that the F18 epitope from Mtb’s Acr1 protein promotes IL-10 and Treg responses without triggering pro-inflammatory IL-6, this suggests a potential immunomodulatory function, the clinical relevance of which in tuberculosis infection merits further investigation [6]. Studies suggest that endometrial micropolyps may represent a hysteroscopic feature of ETB [7]. Infertile women with a history of tuberculosis, hysteroscopy combined with histopathological examination is essential for evaluating endometrial status [8]. Since ETB can only be diagnosed through endometrial biopsy, it often remains undetected. Poor medication adherence has led to an increasing incidence of drug-resistant tuberculosis, and some drug resistance may also be associated with variations in certain genes, although many new drugs are currently under development [9, 10]. However, reports on IVF outcomes in patients with concurrent ETB are scarce and predominantly limited to case reports [11, 12]. ETB may lead to conditions such as hypomenorrhea and intrauterine adhesions, though adequate and full-course anti-tuberculosis therapy has been shown to improve the uterine environment [13, 14].

Recent studies indicate that prompt diagnosis and treatment of NSCE, particularly through hysteroscopy and immunohistochemistry, can significantly improve clinical pregnancy outcomes in women with unexplained infertility. In NSCE patients, resolution after antibiotic therapy is associated with higher pregnancy and live birth rates in subsequent IVF cycles [15]. A retrospective study reported improved natural pregnancy rates after antibiotic treatment for NSCE in women with unexplained infertility [5]. A prospective cohort study further demonstrated that successful antibiotic therapy for NSCE did not significantly increase miscarriage risk in subsequent IVF/ICSI cycles [16]. However, some researchers suggest that the adverse impact of NSCE on reproductive outcomes may be overstated, noting that successful IVF pregnancies and live births can occur even without prior antibiotic treatment [17]. Therefore, this retrospective study aimed to compare clinical outcomes—particularly miscarriage rates following IVF/ICSI-ET between patients diagnosed with ETB and those with NSCE via hysteroscopy, and to explore the potential impact of ETB on reproductive prognosis.

## Materials and methods

### Study population and design

A retrospective, single-center cohort study was conducted on women aged 18–40 years who underwent assisted reproductive technology (ART) treatment at West China Second University Hospital of Sichuan University between January 2019 and December 2024. Patient demographic characteristics and laboratory parameters were obtained from the hospital’s electronic medical record system.

Inclusion criteria were as follows: (1) histopathological diagnosis of ETB or NSCE; (2) Undergoing IVF/ICSI treatment at our center. Exclusion criteria included: (1) isolated pelvic or tubal tuberculosis without endometrial involvement; (2) concurrent major reproductive system anomalies, immune diseases, or endocrine disorders; (3) incomplete follow-up data that could affect outcome assessment. A total of 409 patients meeting the eligibility criteria were enrolled, comprising 27 cases in the ETB group and 382 cases in the NSCE group. All patients underwent hysteroscopy prior to embryo transfer. Diagnosis of ETB was based on histological findings of chronic granulomatous endometritis combined with a positive nucleic acid test for Mycobacterium tuberculosis complex in endometrial tissue. Diagnosis of NSCE was established when histology suggested chronic endometritis without granulomatous changes and immunohistochemistry showed ≥ 5 CD138-positive plasma cells per 10 high-power fields (HPF). Treatment protocols were as follows: patients with NSCE received doxycycline hydrochloride (100 mg twice daily for 14 days), while patients with ETB underwent anti-tuberculosis therapy for 9–12 months before embryo transfer. Patients with NSCE proceeded directly to embryo transfer following medical treatment without undergoing a confirmatory hysteroscopic revaluation. In contrast, all patients with ETB underwent a follow-up hysteroscopy after treatment to confirm microbiological and histological cure prior to embryo transfer.

### Controlled ovarian stimulation protocols

Ovarian stimulation was performed using flexible protocols tailored to individual ovarian reserve and patient characteristics, including the long protocol, short protocol, antagonist protocol, and mild stimulation protocol. The initial dose of gonadotropin (Gn) ranged from 112.5 to 300 IU per day, adjustments were made according to ovarian response. Gonadotropin-releasing hormone (GnRH) agonists or antagonists were routinely administered to prevent premature luteinizing hormone (LH) surges. Triggering with human chorionic gonadotropin (hCG; 5,000–10,000 IU) was performed when at least two follicles reached a diameter of ≥ 18 mm. Oocyte retrieval was conducted 36–38 h after hCG administration [18, 19].

Embryo transfer: whether to perform fresh or frozen was decided based on endometrial thickness and individual clinical circumstances. After treatment for endometritis, patients underwent embryo transfer and were followed up until 12 weeks of gestation. Outcomes including biochemical pregnancy, clinical pregnancy, miscarriage, and live birth rates were recorded to evaluate treatment efficacy and transfer success. The study was approved by the Ethics Committee of West China Second University Hospital.

### Embryo culture and transfer

Embryo transfer was performed on day 3 or day 5 after oocyte retrieval, with high-quality embryos being given priority. For frozen embryo transfer, either a natural cycle or a hormone replacement cycle was utilized. Luteal phase support was initiated following embryo transfer and consisted of progesterone injections and/or vaginal progesterone gel, supplemented with oral dydrogesterone. This regimen was continued until pregnancy was confirmed and maintained for 11–12 weeks thereafter. Serum hCG levels were measured 10–14 days after embryo transfer to assess biochemical pregnancy. Clinical pregnancy and live birth were confirmed via ultrasonography, and any miscarriage events were recorded throughout follow-up.

### Definition of outcomes and statistical analysis

The primary outcomes included clinical pregnancy rate, miscarriage rate, and live birth rate. Clinical pregnancy was defined as the visualization of at least one gestational sac with detectable fetal cardiac activity via ultrasound. Miscarriage was defined as spontaneous pregnancy loss before 12 weeks of gestation. Live birth was defined as the delivery of a live infant at or beyond 24 weeks of gestation.

Normality of continuous variables was assessed using the Shapiro–Wilk test. Normally distributed continuous variables were expressed as mean ± standard deviation, while non-normally distributed variables were presented as median (interquartile range). Comparisons between groups were performed using the two-sided independent t-test or Mann–Whitney U test, as appropriate. Categorical variables were reported as number (percentage) and compared using the chi-square test or Fisher’s exact test.

To evaluate the effect of ETB on laboratory parameters and pregnancy outcomes, generalized estimating equations (GEE) with patient ID specified as the clustering variable were used for embryo-level laboratory outcomes to account for within-patient correlation. For pregnancy outcomes with limited events, Firth’s penalized likelihood logistic regression was performed to reduce potential small-sample bias. Covariates were selected based on previous literature and clinical relevance and included female age, body mass index (BMI), educational level, duration of infertility, type of infertility, infertility cause, controlled ovarian stimulation protocol, and assisted reproductive ART method. To account for multiple comparisons, *P*-values were adjusted using the Benjamini–Hochberg false discovery rate (FDR) correction where applicable. All statistical analyses were performed using SPSS version 26.0, and a two-sided *P*-value < 0.05 was considered statistically significant.

## Results

The baseline characteristics of the study participants are presented in Table 1. No significant differences were observed between the two groups in terms of age, BMI, controlled ovarian stimulation protocol, endometrial thickness or ART method. However, patients in the ETB group had a significantly longer duration of infertility (5 years vs. 2 years, *P* < 0.001) and a higher proportion of primary infertility (81.5% vs. 50.0%, *P* = 0.005). Educational attainment also differed between the groups (*P* = 0.028), with a higher proportion of patients in the ETB group having completed middle school or less. In addition, although not statistically significant, the ETB group showed a trend toward a higher total gonadotropin dose (*P* = 0.059) and a higher likelihood of female-related infertility factors (*P* = 0.056).

**Table 1:** Baseline characteristics

Characteristic	Total (*N* = 409)	Non-endometrial tuberculosis (*n* = 382)	Endometrial tuberculosis (*n* = 27)	*P*-value
Femal’s age, median (Q1-Q3), years	32.00 (29.00, 35.00)	32.00 (29.00, 35.00)	32.00 (30.50, 34.00)	0.962
BMI, median (Q1-Q3)	21.63 (19.98, 23.44)	21.63 (19.98, 23.44)	22.03 (20.09, 23.77)	0.454
Educational level, n(%)				0.028
Middle school or below	96 (23.5)	84 (22.0)	12 (44.5)	
High school/technical secondary school/college	139 (34.0)	133 (34.8)	6 (22.2)	
Undergraduate or above	174 (42.5)	165 (43.2)	9 (33.3)	
Infertility duration, median (Q1-Q3), years	3.00 (1.00, 5.00)	2.00 (1.00, 4.00)	5.00 (3.00, 8.00)	< 0.001
Dosage of gonadotropin, median (Q1-Q3), IU	2325 (1900, 2800)	2312 (1875, 2775)	2700 (2250, 3037)	0.059
Endometrial thickness (single layer) on HCG day (Q1-Q3), mm	5.30 (4.70, 5.95)	5.40 (4.60, 6.00)	5.20 (4.80, 5.90)	0.551
Infertility type, n(%)				0.005
Primary	213 (52.1)	191 (50.0)	22 (81.5)	
Secondary	196 (47.9)	191 (50.0)	5 (18.5)	
Infertility factor, n(%)				0.056
Female factor	284 (69.4)	260 (68.1)	24 (88.9)	
Male factor	43 (10.5)	43 (11.3)	0 ( 0.0)	
Both female and male factors	82 (20.0)	79 (20.7)	3 (11.1)	
COS protocols, n(%)				0.435
GnRH agonist	105 (25.7)	98 (25.7)	7 (25.9)	
GnRH antagonist	282 (68.9)	262 (68.6)	20 (74.1)	
Others	22 ( 5.4)	22 ( 5.8)	0 ( 0.0)	
ART methods, n(%)				0.236
IVF	261 (63.8)	240 (62.8)	21 (77.8)	
ICSI	133 (32.5)	127 (33.2)	6 (22.2)	
Both	15 ( 3.7)	15 ( 3.9)	0 ( 0.0)	

The differences in fertility indicators are shown in Table 2. No significant differences were observed in antral follicle count (AFC) or anti-Müllerian hormone (AMH) levels between the two groups during ovarian reserve assessment. However, patients with ETB had a higher median basal follicle-stimulating hormone (FSH) level (7.8 vs. 6.7 mIU/mL, *P* = 0.032). Regarding ovarian stimulation outcomes, the two groups showed comparable serum estradiol, progesterone, and LH levels on the day of hCG administration. There were also no statistically significant differences in key embryological parameters, including number of oocytes retrieved, number of mature oocytes (MII), and number of normally fertilized oocytes (2PN). Although elevated basal FSH in the ETB group suggests a subtle decline in ovarian function, AMH levels, AFC, and final stimulation outcomes were comparable between the groups. As a result, both groups achieved similar oocyte yield and embryological performance.

**Table 2:** Differences in fertility indicators

	Median (Q1-Q3)			
Variable	Non-endometrial tuberculosis (*n* = 382)	Endometrial tuberculosis (*n* = 27)	Median difference (95% CI)	*P-value*
bFSH, mIU/mL	6.7 (4.5, 8.6)	7.8 (6.1, 9.3)	1.10 (0.10, 1.97)	0.032
AMH, ng/mL	3.0 (1.6, 5.0)	2.6 (1.8, 3.4)	-0.46 (-1.37, 0.23)	0.227
AFC, No.	10.0 (7.0, 14.0)	10 (7.0, 14.0)	0.0 (0.00, 0.00)	0.693
Hormone levels on trigger day				
E2 pg/mL	2641.0 (1741.0, 3793.0)	2600.0 (2004.0, 3369.0)	-40.45 (-371.98, 359.11)	0.972
P, ng/mL	0.9 (0.6, 1.2)	0.9 (0.7, 1.1)	-0.02 (-0.14, 0.16)	0.940
LH, IU/L	1.6 (0.9, 2.7)	1.4 (0.5, 3.4)	-0.20 (-1.00, 0.90)	0.533
Number of oocytes retrieved	10.0 (6.0, 15.0)	9.0 (6.0, 12.0)	-1.0 (-3.00, 3.00)	0.780
Number of mature oocytes	8.0 (5.0, 13.0)	7.0 (5.0, 12.0)	-1.00 (-2.99, 3.50)	0.821
Number of 2PN	6.0 (3.0, 9.0)	6.0 (4.0, 8.0)	0.00 (-2.00, 2.00)	0.999

*bFSH* basal follicular stimulation hormone, *AFC* Antral follicle count, *E2* Estradiol, *P* Progesterone, *LH* Luteinizing hormone

Details of the embryological laboratory parameters are presented in Table 3. No significant differences were observed between the ETB group and the control group across key indicators of embryo development. Specifically, the rates of 2PN fertilization, cleavage, usable and high-quality embryo formation, blastocyst formation, and usable and high-quality blastocyst development. were comparable between the two groups, with all *P* > 0.05.These findings suggest that, despite the presence of ETB, the fertilization competence of oocytes and the early developmental potential of embryos during IVF remained unimpaired. The embryological outcomes in the ETB group were similar to those observed in the NSCE group.

**Table 3:** The impact of endometrial tuberculosis and endometritis on laboratory outcomes

	No.total No. (%)			
Variable	Non-endometrial tuberculosis (*n* = 382)	Endometrial tuberculosis (*n* = 27)	OR (95% CI)	*P-value*
2PN rate			0.99 (0.65, 1.52)	0.968
IVF	1549/2442 (63.4)	156/255 (61.2)		
ICSI	944/1170 (80.7)	29/35 (82.9)		
IVF and ICSI	134/217 (61.8)	0/0 (0.0)		
2PN cleavage rate	2589/2627 (98.6)	183/185 (98.9)	1.05 (0.95, 1.08)	0.256
Available embryo rate	1713/2589 (66.2)	131/183 (71.6)	1.32 (0.74, 2.37)	0.347
Available blastocyst rate	1052/2589 (40.6)	67/183 (36.6)	1.14 (0.69, 1.88)	0.623
Top-quality embryo rate	1160/2589 (44.8)	101/183 (55.2)	1.43 (0.90, 2.27)	0.132
Top-quality blastocyst rate	1074/2589 (41.5)	74/183 (40.4)	1.14 (0.69, 1.89)	0.599
Blastocyst formation rate	1150/2589 (44.4)	77/183 (42.1)	1.10 (0.66, 1.82)	0.724

Clinical pregnancy outcomes of the ETB group and the NSCE group are detailed in Table 4. After excluding cancelled transfer cycles, 151 NSCE patients remained in the final outcome analysis. No statistically significant differences were observed between the two groups in terms of live birth rate (30.0% vs. 42.4%, *P* = 0.699) or clinical pregnancy rate (51.9% vs. 49.0%, *P* = 0.769). However, the miscarriage rate was significantly higher in the ETB group compared to the control group (22.2% vs. 6.6%), with an odds ratio (OR) of 10.60 (95% confidence interval [CI]: 2.03–76.94). Patients with ETB achieved a clinical pregnancy rate comparable to that of the control group. However, they faced a significantly increased risk of miscarriage after conception, contributing to a lower live birth rate in this cohort.

**Table 4:** The impact of endometrial tuberculosis and endometritis on pregnancy outcomes

	No.total No*. (%)			
Variable	Non-endometrial tuberculosis (*n* = 151)	Endometrial tuberculosis (*n* = 27)	OR (95% CI)	*P-value*
Biochemical pregnancy rate	94/151 (62.3)	14/27 (51.9)	0.95 (0.38, 2.40)	0.977
Clinical pregnancy rate	74/151 (49.0)	14/27 (51.9)	1.47 (0.59, 3.73)	0.769
Live birth rate	64/151 (42.4)	8/27 (30.0)	0.78 (0.29, 2.03)	0.699
Miscarriage	10/151 (6.6)	6/27 (22.2)	10.60 (2.03, 76.94)	0.075

^*^Outcomes are reported per embryo transfer cycle

## Discussion

The findings of this retrospective study indicate that, despite no significant differences in baseline characteristics and embryological outcomes, patients with a history of ETB exhibited a higher miscarriage rate following IVF-ET compared to those with NSCE. Our study included a comparable number of patients with ETB to that reported in the domestic literature. However, while previous research identified significantly reduced endometrial thickness, fertilization rate, high-quality embryo rate, and implantation rate in ETB patients compared to controls with non-tuberculous tubal infertility, our analysis using a control group of patients with NSCE revealed a distinctly higher miscarriage rate in the ETB group. This discrepancy in clinical outcomes likely reflects the critical influence of the selected control population, highlighting that ETB may affect post-implantation pregnancy sustainability differently than other forms of infectious or non-infectious endometrial pathology [20]. Although ETB may be successfully eradicated through treatment, the resultant structural or functional damage to the endometrium is often long-lasting. Tuberculous infection can lead to endometrial fibrosis, impaired vascularization, and alterations in the local immune microenvironment. Even after the pathogen is cleared, these persistent changes may compromise endometrial receptivity, thereby hindering proper embryo implantation and increasing the risk of early pregnancy loss [8]. Untreated pulmonary tuberculosis is associated with adverse pregnancy outcomes following IVF-ET, particularly among patients with unexplained infertility. This underscores the clinical significance of tuberculosis in this specific patient population [21].

Endometrial receptivity refers to the ability of the endometrium to accept embryo implantation within a specific temporal window. Studies have shown that pathological changes in the endometrium, such as chronic endometritis, may impair endometrial receptivity and, consequently, reduce the success rates of in vitro fertilization [15]. ETB may lead to more severe impairment of endometrial receptivity compared to NSCE. This may be attributed to the more intense inflammatory response triggered by Mycobacterium tuberculosis, along with its more extensive and profound destructive effects on the endometrium. Such pathological changes can disrupt endometrial cell proliferation and differentiation, and interfere with the essential communication mechanisms between the endometrium and the embryo [22]. Miscarriages associated with NSCE are primarily related to the local release of inflammatory factors, infiltration of immune cells, and potential immune rejection of the embryo by the endometrium. In contrast, ETB may further elevate the risk of miscarriage through more extensive tissue damage, such as impaired decidualization, aberrant angiogenesis, or alterations in uterine cavity morphology [23]. Moreover, ETB-induced persistent inflammation can lead to abnormal remodeling of endometrial structure and function, thereby increasing the risk of miscarriage. Inflammation activates the TLR4-NF-κB signaling pathway, elevating pro-inflammatory factors such as TNF-α and IL-6, which disrupt stromal cell function and cause an imbalance in MMP/TIMP. This results in increased collagen deposition and mild fibrosis. Additionally, glandular disorganization, stromal edema, and abnormal microvascular formation impair spiral artery remodeling and local blood perfusion. Concurrently, the expression of decidualization-related molecules such as HOXA10 and LIF decreases, destabilizing the implantation window. These changes collectively weaken embryo attachment and invasion capabilities, leading to insufficient support at the maternal-fetal interface and an increased susceptibility to early miscarriage [24].

The present study found no significant differences in baseline characteristics or embryological parameters between the two groups, which further supports the possibility that ETB acts as an independent risk factor. These findings suggest that patients with a history of ETB may face a higher risk of miscarriage following IVF-ET, even when their general profiles and embryo quality are comparable to those of patients with NSCE. This observation underscores the importance of more comprehensive endometrial evaluation and potential targeted interventions for women with a history of tuberculosis in the context of assisted reproduction, aim to improve pregnancy outcomes. Future large-scale prospective studies with long-term follow-up are warranted to compare patients with different endometrial pathologies, including ETB and NSCE, and to provide a more comprehensive assessment of their effects on IVF-ET outcomes and live birth rates.

### Strengths and limitations

Strengths include clear diagnostic criteria for ETB (histopathology + Mycobacterium tuberculosis nucleic acid testing) and NSCE (histopathology + CD138 immunohistochemistry), comprehensive assessment of key reproductive outcomes and embryological parameters, and multivariate regression confirming ETB as an independent miscarriage risk factor. Limitations: single-center retrospective design with uneven sample sizes, reducing generalizability and statistical power; due to the limited overall sample size and the absence of miscarriage events in the cohort during the mid-to-late pregnancy period, the statistical power for related analyses may be insufficient; wide miscarriage OR confidence interval; and lack of subgroup analyses to clarify ETBs mechanistic impact.

## Conclusions

In patients undergoing IVF/ICSI-ET, ETB is associated with a significantly higher risk of miscarriage compared to non-specific chronic endometritis, despite similar clinical pregnancy rates. This underscores the need for distinct clinical management and counseling in ETB cases.

## Supplementary Information

Supplementary Material 1.

## Data Availability

The datasets used and/or analysed during the current study are available from the corresponding author on reasonable request.

## Abbreviations

AFC: Antral follicle count
AMH: Anti-Müllerian hormone
ART: Assisted reproductive technology
BMI: Body mass index
ETB: Endometrial tuberculosis
FSH: Follicle-stimulating hormone
Gn: Gonadotropin
GnRH: Gonadotropin-releasing hormone
hCG: Human chorionic gonadotropin
HPF: High-power fields
IVF: In vitro fertilization
ICSI-ET: Intracytoplasmic sperm injection-embryo transfer
LH: Luteinizing hormone
NSCE: Non-specific chronic endometritis
RIF: Recurrent implantation failure
