# Supplementary material for: Endometrial tuberculosis increases miscarriage risk after IVF/ICSI compared with non-specific chronic endometritis
Source: BMC Pregnancy Childbirth. 2026 Mar 21;26:470. doi: 10.1186/s12884-026-08960-2 (PMC13126717; doi:10.1186/s12884-026-08960-2)
Supplement: Supplementary file 1 — Supplementary Material 1. [file 12884_2026_8960_MOESM1_ESM.docx]

Table S1. Table of model output for multivariate regression analysis of biochemical pregnancy.

| Variable | OR (95% CI) | *P*-value | VIF |
| --- | --- | --- | --- |
| Biochemical pregnancy rate |  |  |  |
| Femal’s age | 0.92 (0.83-1.01) | 0.448 | 1.236 |
| BMI | 1.07 (0.94-1.21) | 0.783 | 1.081 |
| Educational level |  |  | 1.098 |
| Middle school or below | 1.01 (0.41-2.54) | 0.987 |  |
| High school/technical secondary school/college | 1.47 (0.65-3.39) | 0.783 |  |
| Undergraduate or above | - | - |  |
| Infertility duration | 0.99 (0.88-1.12) | 0.940 | 1.273 |
| Dosage of gonadotropin | 0.99 (0.99-1.01) | 0.781 | 1.173 |
| Infertility type, n(%) |  |  | 1.140 |
| Primary | 0.85 (0.39-1.86) | 0.692 |  |
| Secondary | - | - |  |
| Infertility factor, n(%) |  |  | 1.090 |
| Female factor | 0.32 (0.08-1.07) | 0.448 |  |
| Both female and male factors | 0.78 (0.19-2.99) | 0.721 |  |
| Male factor | - | - |  |
| COS protocols, n(%) |  |  | 1.030 |
| GnRH agonist | 0.50 (0.23-1.07) | 0.080 |  |
| GnRH antagonist | 0.54 (0.02-15.9) | 0.685 |  |
| Others | - | - |  |
| ART methods, n(%) |  |  | 1.052 |
| IVF | 1.08 (0.47-2.48) | 0.856 |  |
| ICSI | 1.12 (0.50-2.56) | 0.759 |  |
| Both | - | - |  |
| bFSH | 0.95 (0.85-1.06) | 0.369 | 1.063 |

Table S2. Table of model output for multivariate regression analysis of Clinical pregnancy.

| Variable | OR (95% CI) | *P*-value | VIF |
| --- | --- | --- | --- |
| Clinical pregnancy rate |  |  |  |
| Femal’s age | 0.92 (0.84, 1.02) | 0.104 | 1.222 |
| BMI | 1.02 (0.90–1.15) | 0.758 | 1.064 |
| Educational level |  |  | 1.103 |
| Middle school or below | 0.86 (0.35–2.11) | 0.747 |  |
| High school/technical secondary school/college | 1.67 (0.77–3.70) | 0.198 |  |
| Undergraduate or above | - | - |  |
| Infertility duration | 1.04 (0.92–1.17) | 0.548 | 1.285 |
| Dosage of gonadotropin | 1.00 (0.99–1.00) | 0.610 | 1.161 |
| Infertility type, n(%) |  |  | 1.136 |
| Primary | 0.89 (0.42–1.90) | 0.765 |  |
| Secondary | - | - |  |
| Infertility factor, n(%) |  |  | 1.104 |
| Female factor | 0.53 (0.16–1.65) | 0.272 |  |
| Both female and male factors | 1.00 (0.29–3.38) | 0.989 |  |
| Male factor | - | - |  |
| COS protocols, n(%) |  |  | 1.031 |
| GnRH agonist | 0.57 (0.27–1.17) | 0.129 |  |
| GnRH antagonist | 0.86 (0.03–24.92) | 0.923 |  |
| Others | - | - |  |
| ART methods, n(%) |  |  | 1.070 |
| IVF | 1.08 (0.48–2.45) | 0.848 |  |
| ICSI | 5.72 (0.67–124.97) | 0.152 |  |
| Both | - | - |  |
| bFSH | 0.94 (0.84–1.05) | 0.298 | 1.059 |

Table S3. Table of model output for multivariate regression analysis of Live birth.

| Variable | OR (95% CI) | *P*-value | VIF |
| --- | --- | --- | --- |
| Live birth rate |  |  |  |
| Femal’s age | 0.93 (0.84–1.02) | 0.134 | 1.208 |
| BMI | 0.92 (0.81–1.04) | 0.204 | 1.050 |
| Educational level |  |  | 1.104 |
| Middle school or below | 0.62 (0.24–1.58) | 0.324 |  |
| High school/technical secondary school/college | 1.72 (0.78–3.82) | 0.182 |  |
| Undergraduate or above | - | - |  |
| Infertility duration | 1.10 (0.98–1.25) | 0.119 | 1.293 |
| Dosage of gonadotropin | 1.00 (0.99–1.00) | 0.291 | 1.155 |
| Infertility type, n(%) |  |  | 1.131 |
| Primary | 1.20 (0.55–2.64) | 0.644 |  |
| Secondary | - | - |  |
| Infertility factor, n(%) |  |  | 1.112 |
| Female factor | 0.48 (0.14–1.53) | 0.219 |  |
| Both female and male factors | 0.62 (0.18–2.09) | 0.439 |  |
| Male factor | - | - |  |
| COS protocols, n(%) |  |  | 1.036 |
| GnRH agonist | 0.70 (0.33–1.46) | 0.337 |  |
| GnRH antagonist | 0.80 (0.03–22.77) | 0.880 |  |
| Others | - | - |  |
| ART methods, n(%) |  |  | 1.086 |
| IVF | 1.36 (0.59–3.29) | 0.477 |  |
| ICSI | 3.97 (0.52–36.90) | 0.186 |  |
| Both | - | - |  |
| bFSH | 0.96 (0.85–1.08) | 0.513 | 1.057 |

Table S4. Table of model output for multivariate regression analysis of Miscarriage.

| Variable | OR (95% CI) | *P*-value | VIF |
| --- | --- | --- | --- |
| Miscarriage |  |  |  |
| Femal’s age | 0.99 (0.82–1.20) | 0.938 | 1.369 |
| BMI | 1.43 (1.12–1.90) | 0.008 | 1.298 |
| Educational level |  |  | 1.122 |
| Middle school or below | 2.16 (0.46–10.93) | 0.333 |  |
| High school/technical secondary school/college | 1.23 (0.19–7.59) | 0.819 |  |
| Undergraduate or above | - | - |  |
| Infertility duration | 0.78 (0.57–1.01) | 0.073 | 1.619 |
| Dosage of gonadotropin | 1.00 (0.99–1.01) | 0.514 | 1.137 |
| Infertility type, n(%) |  |  | 1.212 |
| Primary | 0.26 (0.05–1.22) | 0.098 |  |
| Secondary | - | - |  |
| Infertility factor, n(%) |  |  | 1.096 |
| Female factor | 0.56 (0.25–1.65) | 0.994 |  |
| Both female and male factors | 0.87 (0.50–2.13) | 0.856 |  |
| Male factor | - | - |  |
| COS protocols, n(%) |  |  | 1.023 |
| GnRH agonist | 0.59 (0.16–2.21) | 0.412 |  |
| GnRH antagonist | 0.74 (0.46-2.53) | 0.998 |  |
| Others | - | - |  |
| ART methods, n(%) |  |  | 1.093 |
| IVF | 0.49 (0.10–2.63) | 0.377 |  |
| ICSI | 2.85 (0.11–40.26) | 0.455 |  |
| Both | - | - |  |
| bFSH | 0.94 (0.76–1.13) | 0.530 | 1.097 |
